# Molecular basis of Spns2-facilitated sphingosine-1-phosphate transport

**DOI:** 10.1038/s41422-023-00908-x

**Published:** 2023-12-20

**Authors:** Bin Pang, Leiye Yu, Tong Li, Haizhan Jiao, Xiaomei Wu, Jinxin Wang, Ruiping He, Yurou Zhang, Juan Wang, Hongli Hu, Wei Dai, Li Chen, Ruobing Ren

**Affiliations:** 1 Shanghai Key Laboratory of Metabolic Remodeling and Health, Institute of Metabolism and Integrative Biology, Fudan University, Shanghai, China; 2 Kobilka Institute of Innovative Drug Discovery, School of Medicine, the Chinese University of Hong Kong, Shenzhen, Guangdong China; 3 School of Pharmacy, Second Military Medical University, Shanghai, China; 4 Shanghai Qi Zhi Institute, Shanghai, China

**Keywords:** Cryoelectron microscopy, Lipid signalling

Dear Editor,

As one of the critical sphingolipid metabolites in eukaryotes, sphingosine-1-phosphate (S1P) acts as a bioactive lipid mediator in the immune and vascular systems. S1P prompts its physiological roles through two mechanisms, binding to its intracellular targets or extracellular secretion. Intracellular S1P promotes cellular proliferation, whereas plasma S1P facilitates immune cell trafficking, regulates angiogenesis, and helps to maintain vascular integrity.^1^ Due to the amphipathic property, S1P cannot diffuse freely but has to be transported across the cell membrane through active transport.^1^ In the past two decades, several S1P transporters have been identified, including two major facilitator superfamily (MFS) members: Spinster homolog 2 (Spns2) and Mfsd2b, and some ATP-binding cassette family transporters. Among these transporters, Spns2 is the first identified and the most extensively studied.^2^ Here, we reported two cryo-electron microscopy (EM) structures of human Spns2 in inward-open conformations bound to S1P or inhibitor 16d. We also established a cell-based S1P efflux assay (Supplementary information, Fig. S1) and an in vivo assay by evaluating the heart development in zebrafish.^2–5^ The deficiency of Spns2 caused a two-heart phenotype in zebrafish (Fig. 1a). Combining the structural information and functional analysis, we performed extensive mutagenesis studies to decipher key residues involved in S1P recognition and conformational changes of Spns2 through S1P transport. Our study is of great importance in providing insights into the transport mechanism of S1P and guiding rational drug design.

**Fig. 1:**
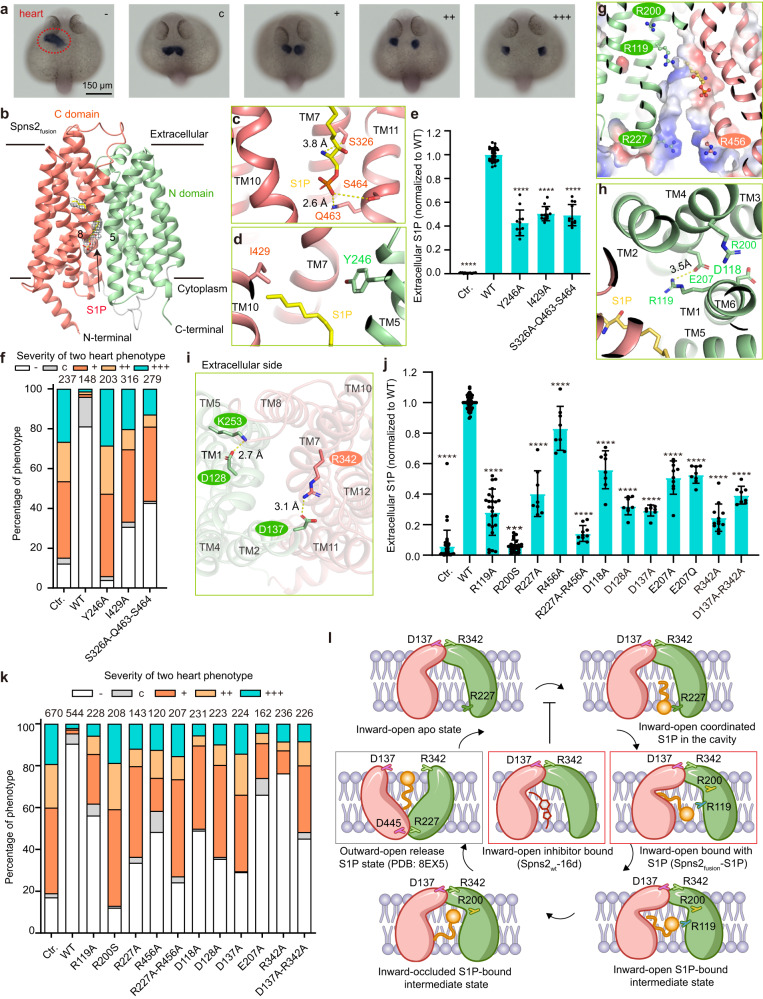
Cryo-EM structure of S1P bound Spns2 and biochemical characterization of Spns2-mediated S1P transport. **a** Deficiency of Spns2 induced two-heart phenotypes of zebrafish. “–” represents the normal heart phenotype; “c”, “+”, “++”, and “+++” represent the severity levels of two-heart phenotypes. **b** Inward-open structure of Spns2_fusion_. TMs of the NTD and CTD are colored pale green and salmon, respectively. The long flexible cytoplasmic loop (P282–S301) between NTD and CTD is colored gray. An elongated density in the central cavity is shown in mesh and fitted with an S1P molecule. S1P is shown as sticks and colored yellow. **c**, **d** Residues (S326, Q463, S464) coordinated the hydrophilic group of S1P in the structure of Spns2_fusion_. Residues (Y246 and I429) interact with the tail of S1P. S1P is shown as **b**. **e** S1P transport assay of WT Spns2 and Y246A, I429A, and S326A-Q463A-S464A mutants. Data are presented as mean ± SD. *n* ≥ 6 biological replicates; ****P < 0.0001. **f** Statistics of two heart ratios of the WT Spns2 and Y246A, I429A, and S326A-Q463A-S464A mutants in the zebrafish two-heart rescue assay. The different two-heart phenotypes are counted and referred to as **a**. The severity levels of two-heart phenotypes are shown in different colors. The total embryo numbers of each group are labeled at the top of each histogram. **g** Four positively charged residues (R227, R456, R119, and R200) line up in the central cavity. Residues are shown as sticks. A cut view of the central cavity with electrostatic potential is displayed. **h** Residues include D118, R119, R200, and E207 on the NTD side. Residues are shown as sticks. R119 interacts with E207 via a salt bridge labeled by a dashed line. **i** Critical interactions at the extracellular side of Spns2 in the inward-open structure. A salt bridge formed between D137 and R342, and interaction between D128 and K253 stabilize the close state of the extracellular side. Residues are shown as sticks. **j** S1P transport assay of WT Spns2 and different mutants. Data are presented as mean ± SD. *n* ≥ 4 biological replicates, ****P < 0.0001. **k** Statistics of two heart ratios of the WT Spns2 and mutants in the zebrafish two-heart rescue assay shown as **f**. **l** Schematic of Spns2-mediated S1P transport cycle. The NTD and the CTD are colored pale green and salmon, respectively. Key residues are displayed and labeled. S1P is colored yellow. Two presented structures represent two conformations in red boxes. The reported structure (PDB: 8EX5) represents the conformation in the gray box.

Spinster homologs are highly conserved among eukaryotes (Supplementary information, Fig. S2). Human Spns2 is a classical MFS member with twelve transmembrane (TM) helices and a molecular weight of ~58 kDa, which is small for cryo-EM analysis. Therefore, we fused a PGS (*Pyrococcus abyssi* glycogen synthase) protein (UniProt ID: Q9V2J8) between F222 and T223 on intracellular loop 2 of Spns2 (named Spns2_fusion_) to enlarge the protein size to facilitate cryo-EM structure determination (Supplementary information, Fig. S3a). Notably, Spns2_fusion_ was mainly expressed in the plasma membrane and maintained a similar S1P transport activity as the wild-type (WT) Spns2 (Supplementary information, Fig. S3b, c). Besides, Spns2_fusion_ presented monodispersed behavior in solution after affinity and chromatography purifications (Supplementary information, Fig. S3d). We determined the structure of the Spns2_fusion_ construct at an overall resolution of 3.60 Å using cryo-EM (Supplementary information, Fig. S3e–k and Table S1). Notably, an elongated extra density in the central cavity extends into the C-terminal domain (CTD) (Fig. 1b). This density could accommodate one S1P molecule well (Fig. 1b; Supplementary information, Fig. S3k). The affinity selection mass spectrometry results confirmed that endogenous S1P bound to Spns2_fusion_ (Supplementary information, Fig. S4).

The Spns2 adopts an inward-open conformation composed of the N-terminal domain (NTD, including TM1–6) and CTD (including TM7–12). These two domains are linked by a long flexible cytoplasmic loop and a short intracellular helix 1 (Fig. 1b; Supplementary information, Fig. S5a). A large intracellular amphiphilic cavity was observed between the NTD and CTD of Spns2 (Supplementary information, Fig. S5b).

The phosphoryl group of S1P forms extensive polar interactions with Spns2, specifically residues Q463 and S464 on TM11, and the amino group of S1P interacts with S326 on TM7 (Fig. 1c; Supplementary information, Fig. S5c). The alkyl tail of S1P is inserted into the pocket mainly composed of hydrophobic residues on TM7–10, and several residues on TM1–2 and TM5 surround S1P on the NTD side (Fig. 1d; Supplementary information, Fig. S5d, e). Given the above structural analysis, we made mutants to validate the importance of these residues by the S1P transport assay. Compared to WT Spns2, the double and triple mutants of S326, Q463, and S464, as well as alanine substitutions of Y246 and I429, substantially decreased S1P transport activity, but other residues showed less effect (Fig. 1e; Supplementary information, Fig. S5f, g and Tables S2, S3). Furthermore, we carried out the rescue assay in *Spns2* knock-out zebrafish to trace embryonic heart development. Consistent with the S1P efflux assay, alanine substitutions of Y246, I429, and the triple mutant of S326, Q463, and S464 also considerably affected the heart development in zebrafish (Fig. 1f; Supplementary information, Table S2).

Intriguingly, several positively charged residues, including R227, R456, R119, and R200 are located around the cavity but at some distance from the S1P binding site, which may participate in S1P recognition and translocation during the dynamic transport process(Fig. 1g). Residues D118, R119, R200, and E207 form a hydrogen-bond network to stabilize the local conformation of NTD (Fig. 1h). Furthermore, the salt bridges between D137–R342 and D128–K253, close to the extracellular side, may also play a crucial role in stabilizing the inward-open conformation of Spns2 (Fig. 1i; Supplementary information, Fig. S5h). These residues are highly conserved between human Spns2 and Spns1, a transporter mediating lyso-phospholipid transport from lysosomes (Supplementary information, Fig. S5i).^6^ It indicates the conserved transport mechanism between Spns2 and Spns1. It is worth noting that R119A, R227A, and R227A-R456A substitutions dramatically decreased the S1P transport activity (Fig. 1j). In addition, variants of D118A, D128A, D137A, E207A, E207Q, and R342A retained approximately half of the transport activity (Fig. 1j). These results demonstrated the critical roles of R119, R227, D128A, D137A, and R342 for the S1P transport activity of Spns2. Consistent with the S1P efflux assay, most of these mutants also considerably affected the heart development in zebrafish, except for R119A and R342A variants, showing fewer effects for rescuing the two-heart phenotypes (Fig. 1k; Supplementary information, Table S2). Additionally, we also confirmed that R119A, D128A, Y246A, I429A, R227A-R456, and D137A-R342A substitutions also significantly decreased FTY720-P transport activity, which indicates a similar FTY720-P transport mechanism as S1P (Supplementary information, Fig. S5j and Table S4). The expression level of the mentioned mutants was verified by western-blot analysis and immunofluorescence detection of localization (Supplementary information, Fig. S6).

Previously, the reported Spns homolog from *Hyphomonas neptunium*, HnSpns, shares an 18% sequence identity with human Spns2 (Supplementary information, Fig. S2). The reported structure of HnSpns also adopted an inward-open conformation, similar to human Spns2 (Supplementary information, Fig. S7).^7^ Moreover, several MFS family lipid transporter structures have been reported, such as human LPC transporter Mfsd2a.^8–12^ Given the amphiphilic characteristics of S1P and LPC, we compared the Spns2_fusion_ structure with the structure of Mfsd2a (PDB ID: 7MJS). Notably, the hydrophobic tail of LPC also buries into the narrow pocket of Mfsd2a CTD, as the pocket we observed similarly in the Spns2_fusion_ structure (Supplementary information, Fig. S8).

Due to the biological significance of S1P, pharmacologically targeting S1P transporters would be a potential therapeutic strategy for anti-angiogenic and anti-lymphangiogenic purposes in cancer and auto-immune disease treatments by the inhibition of “inside-out signaling” of the Sphk-S1PR axis. Previously, the Webster group reported an S1P analog 16d as an Spns2 competitive inhibitor.^3^ Here, we determined the WT Spns2 structure (named Spns2_wt_, without PGS fusion) bound with the inhibitor 16d (IC_50_ = 1.93 μM) using cryo-EM at an overall resolution of 3.5 Å (Supplementary information, Fig. S9). The structure of Spns2_wt_ also adopted an inward-open conformation, which is almost identical to the Spns2_fusion_ structure (Supplementary information, Fig. S10a). Although the density for inhibitor 16d is discontinuous and challenging for model building, we roughly estimated that inhibitor 16d occupied the S1P binding pocket (Supplementary information, Fig. S10b). Moreover, we could only measure its inhibition for S1P transport in the cell-based efflux assay when we overexpressed Spns2 without adding exogenous Sph in the medium (Supplementary information, Fig. S10c). We speculated that the high concentration of exogenous S1P will cover up the Spns2 inhibition by 16d, consistent with the reported weak binding efficacy. Webster group also reported a new Spns2 inhibitor 33p with higher IC_50_ (94 nM).^4^ The IC_50_ of 33p in our MS-based assay was 1.198 μM, which is slightly more potent than 16d (Supplementary information, Fig. S10d). The inhibition mechanism of this new inhibitor is worth further investigation.

Recently, the Lee group reported the human Spns2 structures in multiple conformations, including S1P-/16d-bound inward-facing, outward-facing apo, and two outward-facing partially occluded apo structures.^13^ The overall structures of the first two conformations are similar to our results, indicating the reliability of structure determination (Supplementary information, Fig. S11a, b). Notably, compared with S1P bound structure (PDB code: 8EX4), the head group of S1P binds to a different site in our structure, and the phenyl ring of inhibitor 16d shows slightly different conformation (Supplementary information, Fig. S11b, c). Additionally, in the reported outward-open model, the interaction between R227^TM5^ and D445^TM10^, as well as R456^TM11^ and D220^TM4^, may be critical for stabilizing the outward-open conformation (Supplementary information, Fig. 11d). Notably, our transport assay showed that substitution of D445A lost 60% of the S1P transport activity (Supplementary information, Fig. S11d).

Previously, the Spns2 homolog, HnSpns, is reported as a proton-coupled symporter.^14^ The proposed proton coupling residues of HnSpns, D41 and E129, are identical in human Spns2 (Supplementary information, Fig. S7b). However, the Lee group proposed a distinct mechanism that the human Spns2 is a facilitated-diffusion uniporter. Our mutagenesis study showed that the substitution of E207Q in human Spns2, which is the corresponding residue of E129 in HnSpns, to mimic the protonated state of E207 decreased half of the transport activity in the cell-based assay (Fig. 1j). For further verification of the mechanism, we applied the cell-based transport assay in different pH conditions. Notably, extracellular S1P levels show a pH-dependent manner, favoring the proton-coupled mechanism (Supplementary information, Fig. S12a). We also measure the effects of cations by using K^+^ to replace Na^+^. The results show that the K^+^ group has a slight difference but larger variations compared with the Na^+^ group and the control group (HBSS) (Supplementary information, Fig. S12b).

In summary, we reported two inward-open structures of human Spns2, bound with S1P and the inhibitor 16d, respectively. Using the cell-based efflux assay and the in vivo embryonic heart development assay in zebrafish, we validated the S1P binding site in our structure and identified critical residues for S1P transport through Spns2. The positively charged residues R227 and R119 are critical for S1P transport and may serve as the “holder” to facilitate the phosphoryl group of S1P to flip from the intracellular to the extracellular side. The disease related residue R200 may play a vital role in stabilizing the NTD conformation and the Ser substitution of R200 caused abnormal Spns2 translocation to the plasma membrane. The extensive and dynamic hydrogen-bond network among those charged residues may also stabilize the specific conformations during the S1P transport cycle. Notably, the D137–R342 pair may function as an inward-facing locker, but the R227–D445 pair may serve as an outward-facing locker. Thus, we proposed an alternating access cycle of S1P transport facilitated by Spns2 (Fig. 1l). Besides, we also proposed FTY720-P transport by Spns2 using a similar mechanism as S1P. Altogether, our structural and functional studies of S1P transport shed light on the S1P transport mechanism by Spns2 and will promote the optimization or exploration of new chemical scaffolds of Spns2 inhibitors.

### Supplementary information

Supplementray information

## Data Availability

All data produced or analyzed in this study are included in the main text or the supplementary materials. Cryo-EM maps of Spns2_fusion_ and Spns2_wt_ have been deposited in the Electron Microscopy Data Bank under accession codes: EMD-36284 and 36285, respectively. Atomic models of S1P bound Spns2_fusion_ and 16d bound Spns2_wt_ have been deposited in the Protein Data Bank under accession codes: 8JHQ and 8JHR, respectively.
