# Supplementary material for: Molecular basis of Spns2-facilitated sphingosine-1-phosphate transport
Source: Cell Res. 2023 Dec 20;34(2):173–6. doi: 10.1038/s41422-023-00908-x (PMC10837133; doi:10.1038/s41422-023-00908-x)
Supplement: Supplementary file 1 — Supplementray information [file 41422_2023_908_MOESM1_ESM.pdf]

Supplementary information

Molecular basis of Spns2 facilitated sphingosine-1-phosphate transport

Bin Pang[1,2†], Leiye Yu[1,2†],Tong Li[1†], Haizhan Jiao[2], Xiaomei Wu[1], Jinxin Wang[3], Ruiping He[1], Yurou Zhang[1], Juan Wang[1], Hongli Hu[2], Wei Dai[1*], Li Chen[1,4*], Ruobing Ren[1,4*]

[1] Shanghai Key Laboratory of Metabolic Remodeling and Health, Institute of Metabolism and Integrative Biology, Fudan University, Shanghai, China

[2] Kobilka Institute of Innovative Drug Discovery, School of Medicine, the Chinese University of Hong Kong, Shenzhen, Guangdong 518172, China

[3] School of Pharmacy, Second Military Medical University, Shanghai 200433, China.

[4] Shanghai Qi Zhi Institute, Shanghai 200030, China

* To whom correspondence should be addressed. Email: renruobing@fudan.edu.cn, chenli_imib@fudan.edu.cn, and wei_dai@fudan.edu.cn.

† These authors contribute equally

Materials and methods

Cloning, expression, and purification

The cDNA of human Spns2 (UniProt ID: Q8IVW8) was purchased from Sino Biological (Cat: HG24134-UT) and cloned into pFastBac vector (Invitrogen) with amino-terminal 10 × His tag. Baculovirus was developed with the Bac-to-bac system (Invitrogen) and used for infecting *Spodoptera frugiperda* (Sf9) cells (Union-Bio) at an density of 2 × 10^6 cells per mL and 10 mL of virus per liter of cells. $Spns2_{fusion}$ and all mutations were cloned into pcDNA3.1 vector with amino-terminal 1 × Flag tag. All gene fragments were generated with a standard PCR-based strategy. $Spns2_{fusion}$ was expressed in HEK293F cells. Cells were collected after 60 hours by centrifugation, frozen in liquid nitrogen, and stored at −80 °C.

For $Spns2_{fusion}$, mammalian cell (HEK293F) membrane fractions were homogenized in 20 mL solubilization buffer (25 mM Tris-HCl pH 8.5, 150 mM NaCl, 5% v/v glycerol, 1%DDM), the sample was clarified by ultracentrifugation, and the supernatant was batch bound to an anti-FLAG M2 affinity gel. The resin was washed with buffer containing 25 mM Tris-HCl, pH 8.5, 150 mM NaCl, 0.02% GDN, and eluted in wash buffer containing 0.25 mg/mL^{-1} FLAG peptide. The protein was applied to size-exclusion chromatography on a Superose 6 Increase 10/300 GL column (GE Healthcare) in gel filtration buffer (25 mM Tris-HCl pH 8.5, 150 mM NaCl, 0.01% w/v GDN, 5 mM DTT). Fractions containing the highest concentration of $Spns2_{fusion}$ were collected and concentrated for Cryo-EM.

For $Spns2_{wt}$, cell pellets were disrupted using Dounce, a tissue grinder (homogenizer) for 60 cycles on ice with lysis buffer (25 mM Tris-HCl pH 7.0, 150 mM NaCl, 5% v/v glycerol, 10 μM 16d) and subjected to ultracentrifugation at 150,000 × g for 60 min at 4 °C. The supernatant was discarded, and the membrane fractions were homogenized in 20 mL solubilization buffer (25 mM Tris-HCl pH 7.0, 150 mM NaCl, 5% v/v glycerol, 10 μM 16d) with cocktail inhibitors (Sigma) per initial liter of culture volume. Membrane fractions were dissolved by adding 1% lauryl maltose neopentyl glycol (Anatrace) and 0.1% cholesteryl hemisuccinate (CHS, Sigma-Aldrich) and rotated at 4 °C for 2 h. Cell debris was pelleted at 40,000 × g for 30 min at 4 °C, and the

supernatant was loaded on Ni^{2+}-nitrilotriacetate affinity resin (Ni-NTA, Qiagen), 0.5 mL resin per liter cell culture. The resin was washed twice with 40 column volumes of wash buffer (25 mM Tris-HCl pH 7.0, 150 mM NaCl, 5% v/v glycerol, 30 mM imidazole pH 8.0, 0.01% w/v LMNG, 10 μM 16d), and eluted with elution buffer (25 mM Tris-HCl pH 7.0, 150 mM NaCl, 5% v/v glycerol, 250 mM imidazole pH 8.0, 0.01% w/v LMNG, 10 μM 16d). The protein was applied to size-exclusion chromatography on a Superdex 200 Increase 10/300 GL column (GE Healthcare) in filtration buffer (25 mM Tris-HCl pH 7.0, 150 mM NaCl, 0.005% w/v LMNG, 5 mM DTT, 10 μM 16d). Fractions containing the highest concentration of Spns2 were collected and concentrated for Cryo-EM.

In vitro Cell Based Transport Assay

HEK293T cells (ATCC CRL-11268; mycoplasma free) were co-transfected in a 1.5:0.5 ratio of Spns2 (UniProt ID: Q8IVW8) or mutants: Sphk1 (UniProt ID: Q9NYA1) with polyethyleneimine. Ctr. means control group transfected with empty vector（pCS2 vector）: Sphk1=1.5:0.5. After at least 18 h, transfected cells were harvested and reseeded in 24-well assay plates at a density of 120,000-200,000 cells per well in media (DMEM added 2% dialyzed FBS) and grown to near confluence. Growth media were removed by aspiration, and 0.4 mL of release assay medium (DMEM with 0.2% fatty acid-free BSA) supplemented with 4-deoxypyridinoline (to 1 mM), NaF (2 mM), and Na_3VO_4 (0.2 mM) to retard degradation of S1P by S1P lyase and S1P phosphatases were added to each well. Sphingosine or FTY720 was introduced into duplicate wells (to 5 μM), and plates were placed in a tissue culture incubator for 3 h. Following this incubation, the medium was collected; after centrifugation, 100 μL medium was added to 400 μL of acetonitrile/methanol/formic acid mixture (80:80:1), and the mixture was held at -80°C overnight. After further centrifugation, 0.1 mL of the supernatant fluid was added to UPLC vials, and S1P was quantified by LC-MS by injecting 5 μL into the column.

Time curve of Spns2 transported S1P/FTY720-P

HEK293T cells were transfected with different plasmids in a six-well plate (Sphk1 (UniProt ID: Q9NYA1): Spns2 (UniProt ID: Q8IVW8) =0.5 μg: 1.5 μg, total plasmid: PEI=2 μg: 8 μg). After 24 h incubation (the number of cells was up to nearly 3×10^5), the medium was removed and supplied with 400 μL DMEM with fatty-acid-free BSA (0.2%), 4-deoxypyridoxine (to 1 mM), NaF (to 2 mM), and Na_3VO_4 (to 0.2 mM). After 2 h, 5 μM Sphingosine/FTY720 was added, and 100 μL medium was mixed with 400 μL extract solution at 0.5 h, 1.5 h, 3 h, 6 h, and 9 h. Two replicates were done for each transferred plasmid. The plot was made in GraphPad Prism 8. S1P in the medium in a six-well plate (calculated as S1P produced by 10^6 cells) = 5 μM × response intensity of sample/response intensity of 5 μM standard × 0.1 mL (medium extraction volume) × 20 (total medium volume over actual medium volume in the sample)/ 0.3 (number of cells).

Western blot analysis

The whole HEK293T cell lysate samples were run on SDS-PAGE Gel for 50 min at 200 V. Gels were transferred to PVDF membranes for 90 min at 100 V. The membranes were blocked by 5% (w/v) non-fat milk (1172GR500, BioFroxx) at 4 °C for 1 hour before being sliced into stripes according to a pre-stained ColorMixed Protein Marker. The sliced stripes were incubated with primary antibody at 4 °C overnight. β-actin was used to be a reference protein for Western blot in this project. Then the membranes were washed five times with TBST buffer before incubation with a secondary antibody for 30 min at ambient temperature. The membranes were washed three times and imaged via ChemiScope 6200(Clinx).

S1P measurement by LC-MS

S1P in transfected cells and medium were extracted and measured by LC-MS. LC-MS analysis was performed using a Shimadzu LC system coupled to a TripleTOF mass spectrometer (QTOF 6600+, ABSciex, made in Woodlands Central Industrial Estate, Singapore). Chromatographic separations were obtained under gradient conditions

using a HILIC column (Waters XBridge® Amide, 3.5 μm, 4.6 × 100 mm, made in Ireland). The mobile phase was performed with 20 mM ammonium acetate, 0.1% ammonium hydroxide, and 5 μM ammonium phosphate in 95: 5 water/ACN as phase A and ACN as phase B. The gradient was as follows: 0 min, 85% B; 2 min, 85% B; 3 min, 75% B; 7 min, 75% B; 8 min, 70% B; 9 min, 70% B; 10 min, 50% B; 12 min, 50% B; 12.1 min, 5% B; 15.5 min, 5% B; 15.6 min, 85% B; 21.6 min, 85% B. 5 μL of the extracted samples were injected onto the LC–ESI-MS. The flow rate was set at 0.4 mL/min and the run time at 21.6 min. ESI parameters setup: GS1, 60; GS2, 60; CUR, 40; temperature, 500; ISVF, 5500 or −4500 in positive or negative modes.

The files were converted to the mzXML format using MSConvert software. The mzXML files were then processed by El-MAVEN software (v0.12.0) to generate a peak table. In one experiment batch, two result tables were generated from positive and negative ionization mode detections. In each table, signals of control and all mutant samples were normalized to the reference sample (usually the average of two WT samples). Normalized results from both tables were combined to serve as two technical replicates for each biological sample.

In vivo assay for evaluating Spns2 mutant allele functions in *zebrafish* embryos

pCS2-Spns2 (wild-type and mutant)-P2A-EGFP vectors were constructed using a standard PCR-based strategy. Capped mRNAs were synthesized using mMESSAGE mMACHINE SP6 transcription Kit (Ambion #AM1340). 1 nL of 2.5 μM Cas9 protein (GenScript #Z03470) and 2.5 μM μsgRNA (target site 5'-CAGCGGTCTGGGCTACATCC-3', GenScript #SC1969) [1] to knock out zebrafish Spns2 were injected into wild-type zebrafish embryos at one-cell stage, together with capped RNA expressing control EGFP (10 ng/μL) or human Spns2 wild-type or mutants (250 ng/μL). To validate capped RNA expression, embryos without EGFP signals at 23hpf (hours post fertilization) were removed (less than 8.5%); at 24 hpf, embryos were collected in 4% paraformaldehyde and hybridized to DIG-labeled myl7 riboprobes (primers F: 5'-TGGCTGCATAGATCAGAACCG-3' and R: 5'-TAATACGACTCACTATAGGGGCAGCAGTTACAGACAGAATA-3',

Roche #11277073910) following whole mount in situ hybridization protocols [2]. Two-heart phenotypes were scored according to the scoring system in Figure 1a. To validate the CRISPR editing effect, embryos in the “-” group (normal heart) were collected for gDNA extraction and subject to Sanger sequencing of the Spns2 sgRNA target regions. Each mutant was repeated at least three times.

The IC $_{50}$ measurement of 33p

HEK293T cells were transfected with different plasmids in a six-well plate (Sphk1: Spns2=0.5 μg: 1.5 μg, total plasmid: PEI=2 μg: 8 μg). After 24 h incubation (the number of cells was up to nearly 3 $\times 10^5$), the medium was removed and supplied with 400 μl DMEM with fatty-acid-free BSA (0.2%), 4-deoxypyridoxine (to 1 mM), NaF (to 2 mM), and Na_3VO_4 (to 0.2 mM) with concentration gradient of 33p. After 2 h, 5 μM Sphingosine was added, and 100 μL medium was mixed with 400 μL extract solution (acetonitrile/methanol/formic acid mixture (80:80:1)) at 1 h and precipitated at -80℃ overnight. S1P measured by LC-MS. 33p gradients response normalized to 0 μM 33p response. The plot was made in GraphPad Prism 8. Data analyzed by log(inhibitor) vs. response:Y=Bottom + (Top-Bottom)/(1+10^((X-LogIC50))).

Immunofluorescence

HEK293T cells (ATCCCRL-11268; mycoplasma free) were seeded in a 6-well plate (BIOFIL) at a density of 5×10^5 cells per well. The next day, cells were co-transfected in a 1:1 ratio of transporter: membrane marker EXOC7-GFP with polyethyleneimine. After 24 h post-transfection, the cells were fixed with 4% paraformaldehyde for 10 min at room temperature, permeabilized with 0.2% triton and 0.1% BSA in PBS for 10 min on ice, and blocked with 5% BSA in PBS for 1 h. Cells were then incubated with M2 anti-Flag monoclonal antibody (1:100, Sigma-Aldrich, F1804) overnight at 4°C followed by three times wash with PBS. Cells were then incubated with Alexa Fluor 594 goat anti-mouse IgG secondary antibody (1:200, Thermo Fisher Scientific) for 3 h at 4°C, followed by another three times washed with PBS. Cells were then stained with

DAPI and washed three times with PBS before cover slides were mounted. Images were taken using a Zeiss LSM-780 confocal microscopy.

Native mass spectrometry

$Spns2_{fusion}$ and $Spns2_{wt}$ purified and concentrated to 100 μM (without supplemented with additional S1P) with the lysis buffer 1(25 mM Tris-HCl pH 8.5, 150 mM NaCl, 0.01% w/v GDN) and lysis buffer 2 (25 mM Tris-HCl pH 7.0, 150 mM NaCl, 0.005% w/v LMNG) respectively. 16 μL protein sample added into 60 μL lipid extraction liquid (acetonitrile/methanol/formic acid mixture) and precipitated at -80℃ overnight. S1P measured by LC-MS.

Investigation of Spns2 transport mechanism by mass spectrometry

For pH or cations dependent investigations assay, the transfection cells were reseeded in a 24-well plate at a density of $3x10^5$ cells per well and exchange the culture conditions with different pHs: Hank's balanced salt solution, HBSS, 5 mM HEPES-NaOH pH 7.4 and condition 1 (140 mM NaCl, 20 mM MES pH 6.5, 2 mM $CaCl_2$, 1 g/L D-glucose); condition 2 (140 mM NaCl, 20 mM HEPES-NaOH pH 7.5, 2 mM $CaCl_2$, 1 g/L D-glucose); condition 3 (140 mM NaCl, 20 mM Tris-HCl pH 8.5, 2 mM $CaCl_2$, 1 g/L D-glucose) or different ionic conditions: condition 1 (Hank's balanced salt solution, HBSS); condition 2 (140 mM NaCl, 20 mM Tris-HCl pH 7.5, 2 mM $CaCl_2$, 1 g/L D-glucose); condition 3 (140 mM KCl, 20 mM Tris-HCl pH 7.5, 2 mM $CaCl_2$, 1 g/L D-glucose). Briefly, Growth media were removed by aspiration, and 0.4 mL of condition buffer supplemented with 4-deoxypyridinoline (to 1 mM), NaF (2 mM), and Na_3VO_4 (0.2 mM) and 0.2% fatty acid-free BSA washed the cells and replaced. Sphingosine was introduced into duplicate wells (to 5 μM) and incubated for 3 h. Following this incubation, the medium was collected; after centrifugation, 100 μL medium added 400 μL of acetonitrile/methanol/formic acid mixture (80:80:1), and the

variety was held at -80℃ overnight. After further centrifugation, 0.1 mL of the supernatant fluid was added to UPLC vials, and S1P were quantified by LC-MS.

Cryo-EM sample preparation and data acquisition

For $Spns2_{fusion}$, a total of 3.5 μL of the concentrated protein at 5-10 mg/mL was applied to glow-discharged holey nickel-titanium coated gold grids (CryoMatrix M024-Au300-R12/13, Zhenjiang Lehua Technology) and holey gold supported grids (UltrAufoil Au 300 mesh, R1.2/1.3, Quantifoil). The grids were blotted for 3 s and flash-frozen in liquid ethane using a Vitrobot (Mark IV, Thermo Fisher Scientific). Images were recorded on a 300 kV Titan Krios G3i electron microscope (Thermo Fisher Scientific) equipped with a Gatan K3 Summit direct detector and a GIF Quantum energy filter (slit width 20 eV). Movie stacks were collected using SerialEM[3] in counting mode at a magnification of 105,000x with a corresponding pixel size of 0.85 Å. Movie stacks of $Spns2_{fusion}$ were collected at different times. Each movie stack of $Spns2_{fusion}$ with 50 frames was exposed for 2.5 or 3 s. The dose rate ranges from 14.61 to 17.12 e/px/s, with the total dose range from 50.6 to 59.2 per $Å^2$. The defocus range was set from −1.0 μm to −1.8 μm. A total of 14,396 movie stacks were collected.

For $Spns2_{wt}$, 16d was added into the concentrated protein solutions to a final concentration of 150 μM. A total of 3.5 μL of the concentrated protein at 10 mg/mL was applied to glow-discharged holey carbon-coated gold grids (Quantifiol Au 200 mesh 1.2/1.3) holey gold supported grids (UltraAufoil Au 300 mesh, R1.2/1.3, Quantifoil). The grids were blotted for 3.5 s and flash-frozen in liquid ethane using a Vitrobot (Mark IV, Thermo Fisher Scientific). Images were recorded on a 300 kV Titan Krios G3i electron microscope (Thermo Fisher Scientific) equipped with a Gatan K3 Summit direct detector and a GIF Quantum energy filter (slit width 20 eV). Movie stacks were collected using SerialEM[3] in super-resolution mode at a magnification of 105,000x with a corresponding pixel size of 0.425 Å. Each movie stack of $Spns2_{wt}$ with 50 frames was exposed for 2 s. The dose rate is 18.19 and 18.02 e/px/s, corresponding a total dose of

50.35 and 49.88 per Å^2. The defocus range was set from −1.5 μm to −2.0 μm. A total of 8,640 movie stacks were collected.

Data Processing

For Spns2$_{fusion}$, movie frames were aligned using MotionCor2[4] with five by five patches. Micrograph contrast transfer function (CTF) estimations were performed by CTFFind4[5] using micrographs without dose-weighting. The dose-weighted micrographs were used for particle picking and further processing. Particles were automatically picked using previously produced templates by Gautomatch (https://www.mrc-lmb.cam.ac.uk/kzhang/Gautomatch). 7,607,332 particles of all micrographs were picked. Micrographs with estimated Ctf Max Resolution (> 5 Å) and Ctf Figure Of Merit (> 0.3) were selected for further processing. A total of 13,971 micrographs were selected. A total of 7,397,481 particles were extracted by Relion3.1[6] with a box size of 72 pixels and a pixel size of 3.4 Å. Iterative 2D classifications were performed using cryoSPARC v.2.14.2[7]. A total of 4,032,474 particles were selected and then recentered and re-extracted by Relion with a box size of 144 pixels and a pixel size of 1.7 Å. Twelve parallel ab initio reconstructions were performed in cryoSPARC. A total of 1,055,847 particles from combined good classes were selected. These particles were subjected to iterative 2D classifications, and 976,861 particles were selected. Next, particles were subjected to many rounds of parallel and unparallel ab initio reconstructions, and a more homogeneous particle dataset with 390,253 particles was generated. The parallel ab initio reconstruction is referred to perform ab initio reconstruction with repeated runs (two or more runs with completed same parameters). Then, we select and combine the particles of good classes of repeated runs to proceed to the next step. cryoSPARC will automatically remove the repeated particles from the repeated runs. The unparallel ab initio reconstruction is just the regular run of the job. After one round of ab initio reconstruction and nonuniform refinement, a 3.4 Å map was generated. One round of nonuniform refinement was performed, yielding a 3.4 Å map. Subsequently, particles were recentered and re-extracted by Relion with a box size

of 288 pixels and a pixel size of 0.85 Å. Then, particles were imported into cryoSPARC v.3.3.1. Furthermore, one round of ab inito reconstruction, nonuniform refinement, and local refinement was performed, yielding a 3.60 Å map. Conversions of particle star files between Relion and cryoSPARC were performed mainly using the pyem script[8].

For $Spns2_{wt}$, movie frames were aligned using MotionCor2[4] with five by five patches. Afterward, data processing was performed using cryoSPARC v.3.3.1. Micrographs were imported into cryoSPARC. CTF estimation was performed using Patch CTF. Particles were first picked using a blob picker with partial micrographs. 2D templates were generated by 2D classification. Particle picking of all micrographs was performed by a template picker. Particles were extracted using a box size of 256 pixels and cropped into 128 pixels. After many rounds of 2D classification, two particle sets with 664,888 particles and 2,214,117 particles were selected. For 664,888 particle sets, after one round of ab initio reconstruction and five rounds of heterogeneous refinement, a dataset with 78,714 particles was generated. After that, one round of nonuniform and local refinement was performed. A 3.85 Å map was obtained. For 2,214,117 particle sets, after two rounds of ab initio reconstruction and three rounds of heterogeneous refinement, a dataset with 216,159 particles was generated. Subsequently, two particle sets were combined to do heterogeneous refinement. 237,442 particles were selected. After two rounds of refinement, particle re-extraction, four rounds of ab initio reconstruction, and one round of heterogeneous refinement, a new particle set with 115,145 particles were obtained. Then, one round of ab inito reconstruction, nonuniform refinement, and local refinement was performed, yielding a 3.80 Å map. Furthermore, particles were re-extracted with a box size of 300 pixels. Finally, one round of ab inito reconstruction and nonuniform refinement was performed. A 3.52 Å map was obtained.

Model building and refinement

The initial model was built using the predicted Spns2 model by AlphaFold2 as a

template [9]. Model was fitted into the density map and manually adjusted and rebuilt in COOT[10]. Then, the model was refined in Phenix using real-space refinement with secondary structure and geometry restraints[11] and finally adjusted in COOT. Overfitting of the model was checked by refining the model using one of the two independent maps from gold-standard refinement and calculating FSC against both half maps[12]. The final model was validated using Molprobity integrated into Phenix [13] (Table S1). The FSC between the map and model was calculated by Phenix.mtriage[14]. Structural figures were prepared in PyMOL (https://pymol.org/2/), UCSF Chimera[15], and UCSF ChimeraX[16].

Statistics and reproducibility

Statistical analyses were performed using GraphPad Prism 8 using an ordinary one-way ANOVA for repeat experiments. All results are expressed as mean ± standard deviation (SD) values, n = biological replicates. Differences were considered statistically significant when $p < 0.01$(**); "****" represents $p < 0.0001$. The actual p-value for each test can be found in the supplementary table.

Supplementary References

1 Kotani, H., Taimatsu, K., Ohga, R., Ota, S. & Kawahara, A. Efficient Multiple Genome Modifications Induced by the crRNAs, tracrRNA and Cas9 Protein Complex in Zebrafish. *PLoS One* **10**, e0128319, doi:10.1371/journal.pone.0128319 (2015).

2 Thisse, C. & Thisse, B. High-resolution in situ hybridization to whole-mount zebrafish embryos. *Nat Protoc* **3**, 59-69, doi:10.1038/nprot.2007.514 (2008).

3 Mastronarde, D. N. SerialEM: A Program for Automated Tilt Series Acquisition on Tecnai Microscopes Using Prediction of Specimen Position. *Microscopy and Microanalysis* **9**, 1182-1183, doi:10.1017/S1431927603445911 (2003).

4 Zheng, S. Q. *et al.* MotionCor2: anisotropic correction of beam-induced motion for improved cryo-electron microscopy. *Nat Methods* **14**, 331-332, doi:10.1038/nmeth.4193 (2017).

5 Rohou, A. & Grigorieff, N. CTFFIND4: Fast and accurate defocus estimation from electron micrographs. *J Struct Biol* **192**, 216-221, doi:10.1016/j.jsb.2015.08.008 (2015).

6 Jasenko *et al.* New tools for automated high-resolution cryo-EM structure determination in RELION-3. *eLife* (2018).

7 Punjani, A., Rubinstein, J. L., Fleet, D. J. & Brubaker, M. A. cryoSPARC: algorithms for rapid unsupervised cryo-EM structure determination. *Nat Methods* **14**, 290-296, doi:10.1038/nmeth.4169 (2017).

8 Daniel Asarnow, E. P., & Yifan Cheng. https://doi.org/10.5281/zenodo.3576630 (2019).

9 Tunyasuvunakool, K. *et al.* Highly accurate protein structure prediction for the human proteome. *Nature* **596**, 590-596, doi:10.1038/s41586-021-03828-1 (2021).

10 Emsley, P. & Cowtan, K. J. A. c. s. D. b. c. Coot: model-building tools for molecular graphics. **60**, 2126-2132 (2004).

11 Adams, P. D. *et al.* PHENIX: a comprehensive Python-based system for macromolecular structure solution. **66**, 213-221 (2010).

12 Amunts, A. *et al.* Structure of the yeast mitochondrial large ribosomal subunit. **343**, 1485-1489 (2014).

13 Williams, C. J. *et al.* MolProbity: More and better reference data for improved all-atom structure validation. *Protein science : a publication of the Protein Society* **27**, 293-315, doi:10.1002/pro.3330 (2018).

14 Afonine, P. V. *et al.* New tools for the analysis and validation of cryo-EM maps and atomic models. *Acta crystallographica. Section D, Structural biology* **74**, 814-840, doi:10.1107/s2059798318009324 (2018).

15 Pettersen, E. F. *et al.* UCSF Chimera—a visualization system for exploratory research and analysis. **25**, 1605-1612 (2004).

16 Pettersen, E. F. *et al.* UCSF ChimeraX: Structure visualization for researchers, educators, and developers. **30**, 70-82 (2021).

Supplementary information, note1

SPNS2-mediated S1P and FTY720-P efflux

In cells, sphingosine kinases (Sphks) phosphorylate Sphingosine (Sph) to yield S1P. Fingolimod (FTY720), one of the benchmark drugs to treat relapsing multiple sclerosis (MS), also needs to be phosphorylated by Sphks to FTY720-P, a functional form of S1P receptor agonist (Supplementary information, Fig. S1a). We overexpressed human Sphk1 and Spns2 variants in HEK293 cells. Sph or FTY720 uptaken by cells are phosphorylated by Sphk1 to form S1P or FTY720-P, which are then exported by wild-type Spns2 to the culture medium. Extracellular S1P was extracted with organic solvents and analyzed by LC-MS to calculate the export activity of Spns2 quantitively (Supplementary information, Fig. S1b). As shown, S1P and FTY720-P is accumulated time-dependently if wild-type Spns2 is overexpressed (Supplementary information, Fig. S1c). The previously reported disease related loss-of-function mutation R200S on Spns2 abolished S1P and FTY720-P transport activities (Supplementary information, Fig. S1c). These results confirmed that Spns2 is a transporter of S1P and FTY720-P.

Structure determination

After extensive homolog screenings and crystallization trials, we turned to use cryo-EM to determine the structures of human Spns2. The inserted PGS promotes the initial classification by distinguishing particle qualities. Nevertheless, due to the flexibility of the connected loop between PGS and Spns2, the flexible PGS interferes with the reconstruction to a high resolution. After multiple rounds of 2D and 3D classification using parallel or unparallel ab-initio reconstruction in cryoSPARC, we obtained an EM map of the $Spns2_{fusion}$ construct at an overall resolution of 3.60 Å. The densities for the transmembrane helices of Spns2 were unambiguously determined based on the well-traced α-helices and aromatic side chains (Supplementary information, Fig. S3k). Due to the flexibility, the unclear density of PGS fusion protein can be observed at lower threshold and part of the extra-/intra-cellular loops are invisible.

The wild-type Spns2 structure bound with inhibitor 16d was determined using the detergent of LMNG (Lauryl maltose neopentyl glycol) without binding nanobody or fusing with any fusion partner.

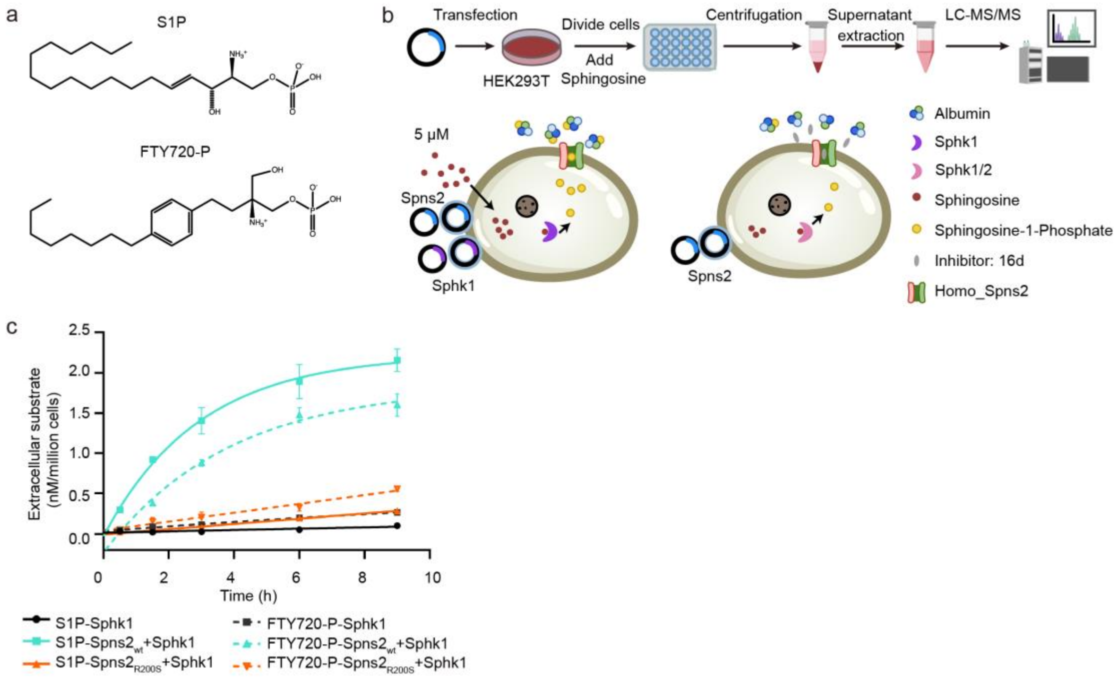

Fig. S1 Cell-based S1P transport assay

a. Structural formulas of S1P and FTY720-P. **b.** Schematic of S1P efflux assay using LC-MS. **c.** Extracellular S1P or FTY720-P accumulations in cell-based efflux assay. S1P concentrations at different time points were measured. Data are presented as mean ± SD (n=4).

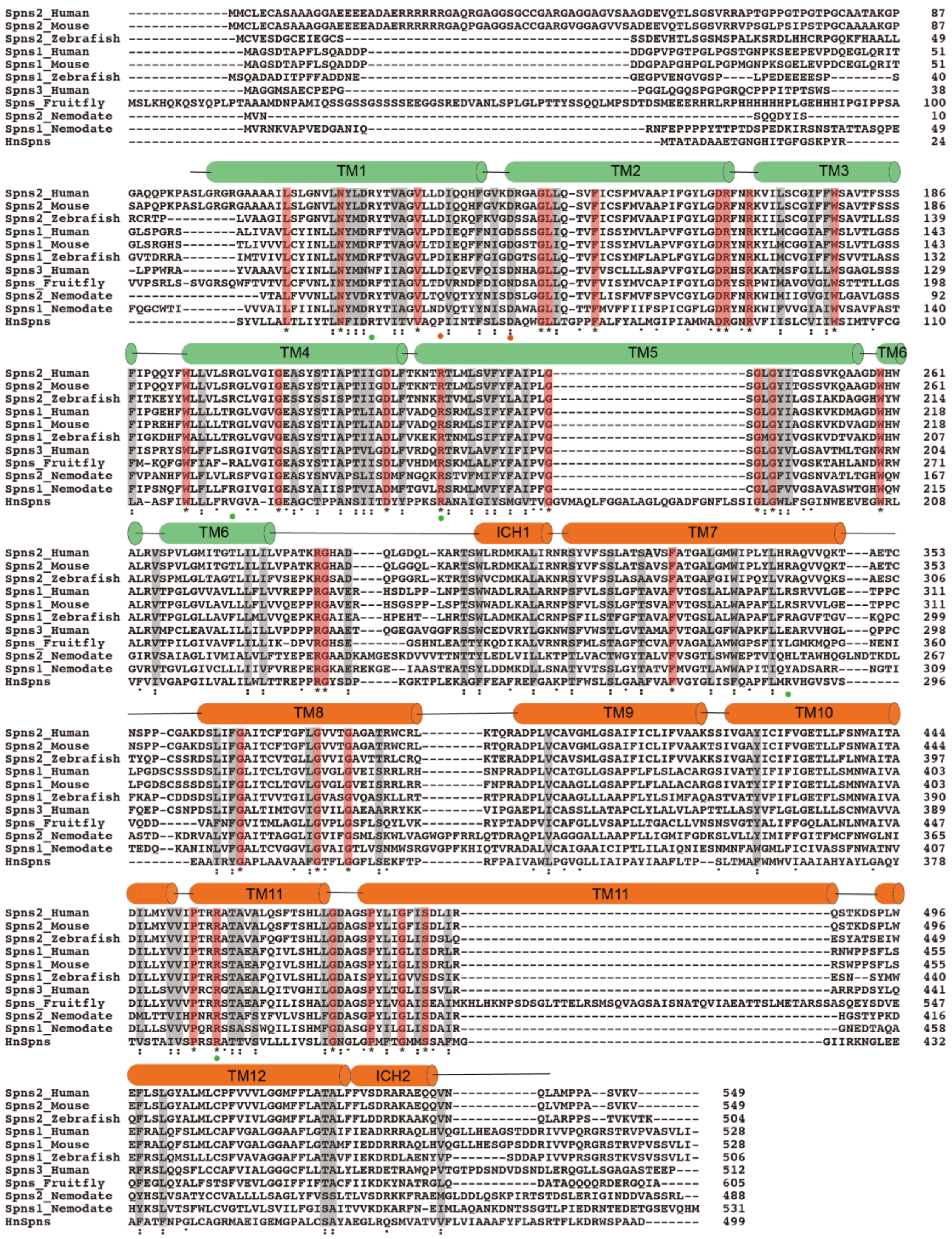

Fig. S2 Sequence alignment of human Spns1-3 with homologs in mouse, zebrafish, fruitfly, nemodate, and *Hyphomonas neptunium*.

Secondary structural elements of human Spns2 are displayed above the alignment. Invariant and highly conserved residues are shaded salmon and gray, respectively. The critical residues identified are indicated by green and salmon circles below.

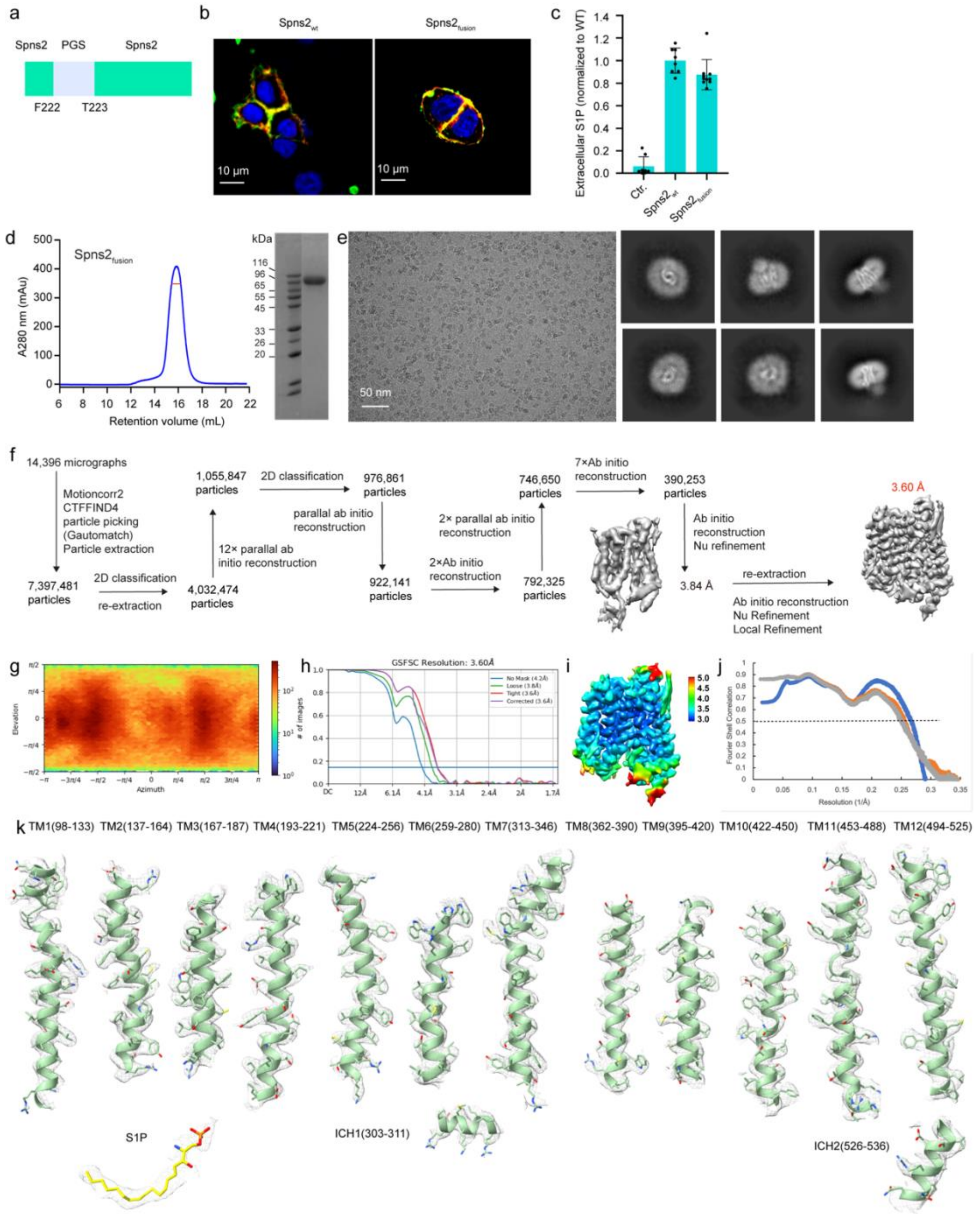

Fig. S3 Structural determination of Spns2$_{fusion}$.

a. Schematic of the insert position of PGS in Spns2. Spns2 and PGS are colored green and gray. **b.** Immunofluorescence localization of Spns2$_{fusion}$ and Spns2$_{wt,}$ both fused with an N-terminal flag tag. Plasma membrane is labeled by GFP (green) fused membrane marker EXOC7. Spns2 is labeled by Alexa Fluor 594 (red) goat anti-mouse IgG secondary antibody. Cell nuclei were stained with DAPI (blue). **c.** Transport assay

of extracellular S1P level when overexpressing the wild-type and fusion form of Spns2 in HEK293T cells. Data are presented as mean ± SD. N≥4. **d.** Spns2$_{fusion}$ purification. Final size exclusion chromatography (Superdex 200 10/300 Increase) elution profile of Spns2$_{fusion}$. SDS-PAGE of one fraction at the elution peak is shown. **e**. Representative micrograph. **f.** Flowchart for EM data processing. Details can be found in the Methods. Representative 2D classes are displayed. **g**. Angular distribution of the particles used for the final reconstructions. **h.** Fourier shell correlation (FSC) curves of the final refined map. **i.** Local resolution of the final refined map of Spns2$_{fusion}$ estimated by cryoSPARC. **j.** Fourier shell correlation (FSC) between map and model of Spns2$_{fusion}$. FSC curve of the final refined model against the full map, colored in blue. FSC curve of the model refined against the first half map against the same map, colored in orange. FSC curve of the model refined against the first half map against the second half map, colored in gray. **k.** The density maps of the transmembrane and intracellular helices of Spns2$_{fusion}$ are shown as mesh at a contour level of 0.2. The density maps of S1P are shown as mesh at a contour level of 0.1.

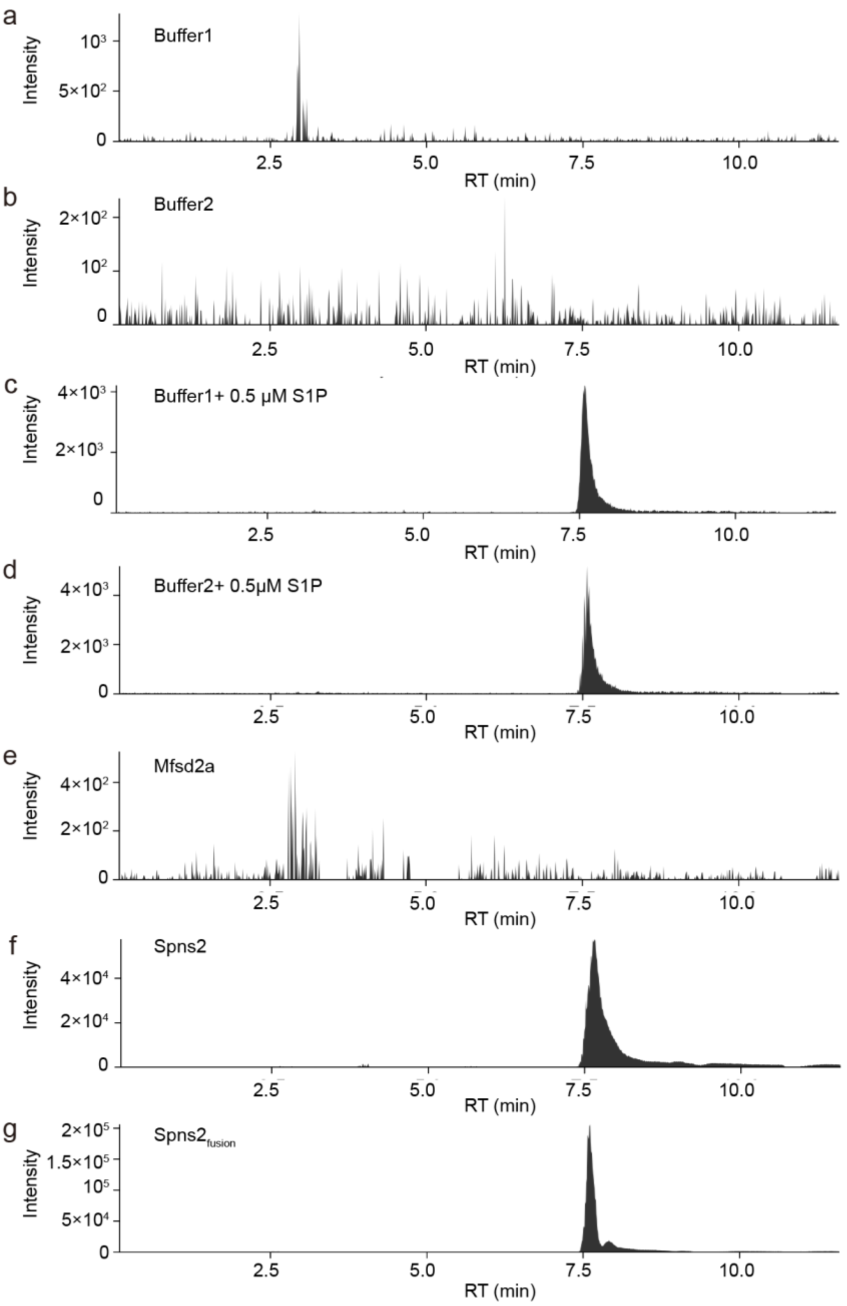

Fig. S4 Identification of S1P in purified Spns2$_{fusion}$ and Spns2$_{wt}$ protein sample by mass spectrometry

a. Buffer1: 25 mM Tris-HCl pH 8.5, 150 mM NaCl, 0.01% w/v GDN. **b.** Buffer2: 25 mM Tris-HCl pH 7.0, 150 mM NaCl, 0.005% w/v LMNG. **c.** Buffer 1 supplemented with 0.5 μM S1P. **d.** Buffer 2 supplemented with 0.5 μM S1P. **e.** 100 μM Mfsd2a in buffer 2. **f.**100 μM Spns2 in buffer 2. **g.**100 μM Spns2$_{fusion}$ in buffer 1.

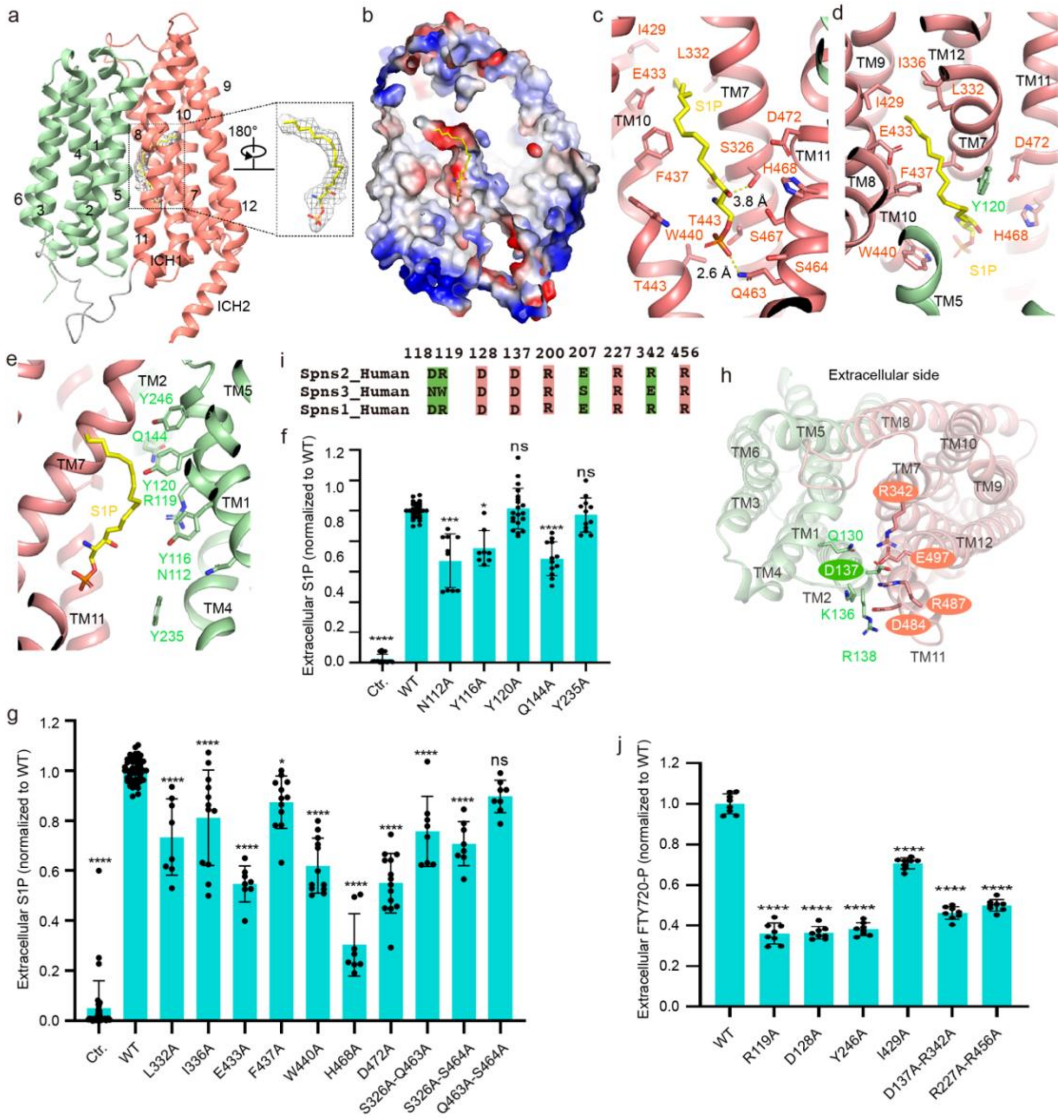

Fig. S5 Structure analysis of Spns2fusion bound with S1P

a. Inward-open structure of Spns2fusion bound with S1P. TMs of NTD and CTD are colored pale green and salmon, respectively. The long flexible cytoplasmic loop (P282-S301) between NTD and CTD is colored gray. S1P density is shown in mesh at a contour level of 0.0697. S1P is shown as sticks and colored yellow. A magnified view of S1P is displayed on the right. **b.** A cut view of the central cavity with electrostatic potential is displayed. A narrow pocket extends into CTD. S1P is shown as sticks and colored yellow. **c**. Residues (S326, Q463, S464, H468, and D472) coordinated the hydrophilic group of S1P in the structure of Spns2fusion. TMs of NTD and CTD are

colored pale green and salmon, respectively. S1P is colored yellow. Residues and S1P are shown in as sticks. **d**. Residues (L332, I336, I429, E433, F437, and W440) interact with of S1P in the structure of $Spns2_{fusion}$. TMs of NTD and CTD are colored pale green and salmon, respectively. S1P is colored yellow. Residues and S1P are shown in as sticks. **e**. Other residues (N112A, Y116A, R119A, Y120A, Q144, Y235, and Y246) surround S1P at the NTD side in the structure of $Spns2_{fusion}$-S1P. **f.** Transport assay of extracellular S1P level when overexpressing Spns2 wild-type or mutations (N112A, Y116A, Y120A, Q144, and Y235A) in HEK293T cells. Data are presented as mean ± SD. n≥4 biological replicates; $p < 0.0001$(****). **g.** Transport assay of extracellular S1P level when overexpressing Spns2 wild-type or mutations (I336A, L332, E433, F437, W440A, H468A, D472A, and three double mutants of S326, Q463, and S464) in HEK293T cells. Data are presented as mean ± SD. n≥4 biological replicates; $p < 0.0001$(****). **h**. Charge network at the extracellular side of the inward-open $Spns2_{fusion}$ -S1P structure. Residues are shown as sticks. **i.** Critical residues alignment among human Spns1, Spns2, and Spns3. **j.** Transport assay of extracellular FTY720-P level when overexpressing Spns2 wild-type or mutations (R119A, D128A, Y246A, I429A, R227A-R456A, and D137A-R342A) in HEK293T cells. Data are presented as mean ± SD. n=4 biological replicates; $p < 0.0001$(****).

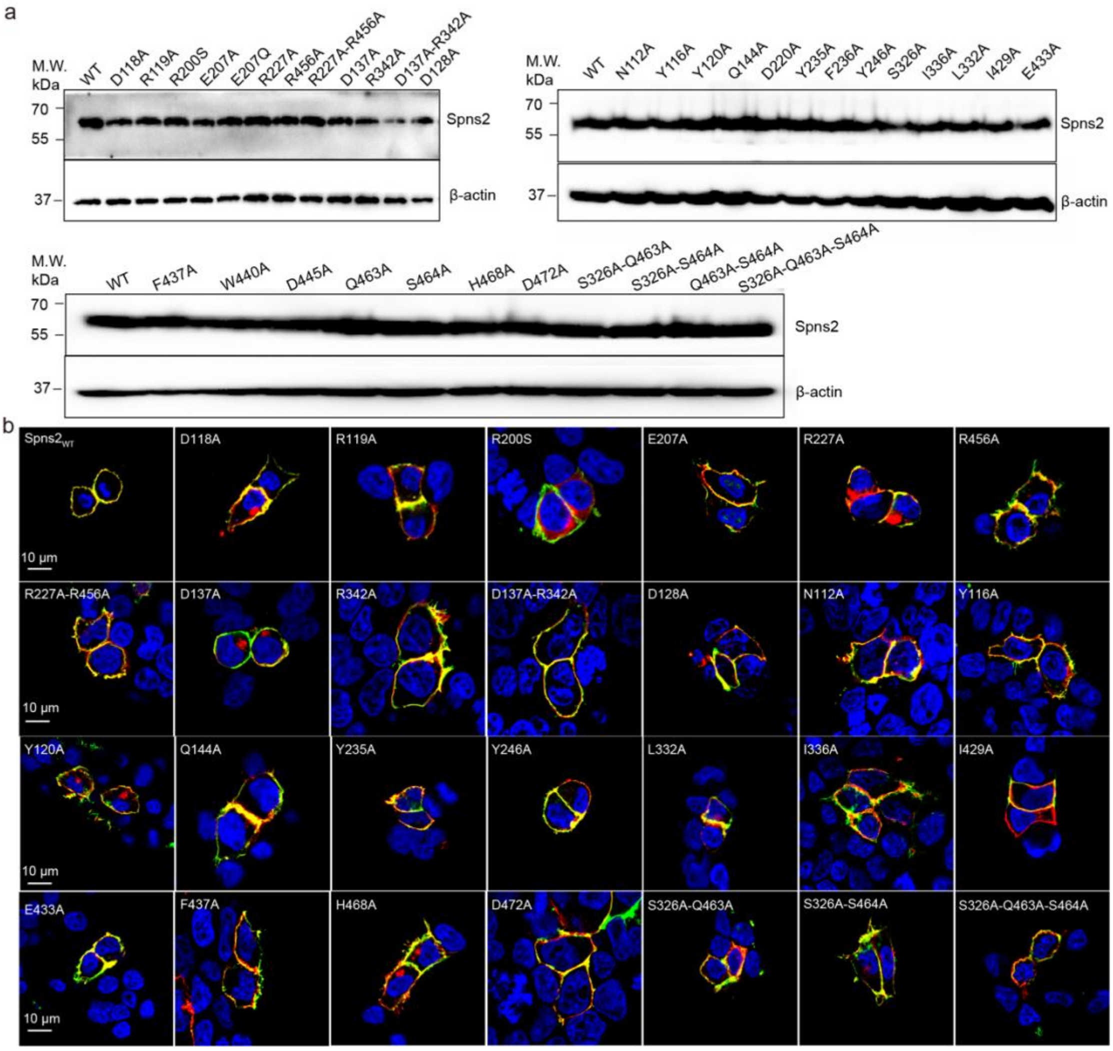

Fig. S6 Expression level and plasma membrane localization of the wild-type and mutants of Spns2

a. Western blot analysis of the expression level of human Spns2 mutants. WT, wild-type. **b.** Plasma membrane localization of Spns2 mutants and wild type by immunofluorescence. Spns2 mutants and the wild-type are all fused with an N-terminal flag tag. Plasma membrane is labeled by GFP (green) fused membrane marker EXOC7. Spns2 is marked by Alexa Fluor 594 (red) goat anti-mouse IgG secondary antibody. Cell nuclei were stained with DAPI (blue).

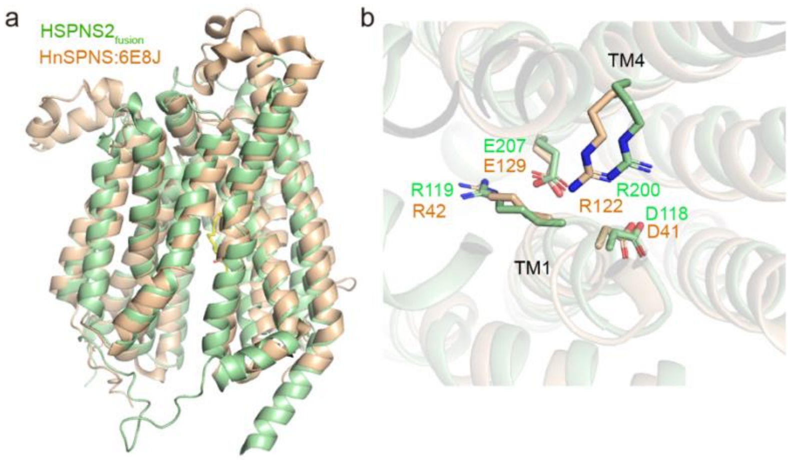

Fig. S7 Structural comparison of Spns2$_{fusion}$ and HnSpns

a. Superposition of human Spns2$_{fusion}$ (pale green) with HnSpns (PDB ID: 6E8J) (wheat**).** The overall root mean-standard deviation (r.m.s.d) between HnSpns and human Spns2 is 3.268 Å over 387 residues majorly located in the TM region. **b.** Residues including D118, R119, R200, and E207 of Spns2$_{fusion}$ (pale green) superimposed with residues including D41, R42, R122, and E129 of HnSpns (wheat**).** Residues are shown as sticks.

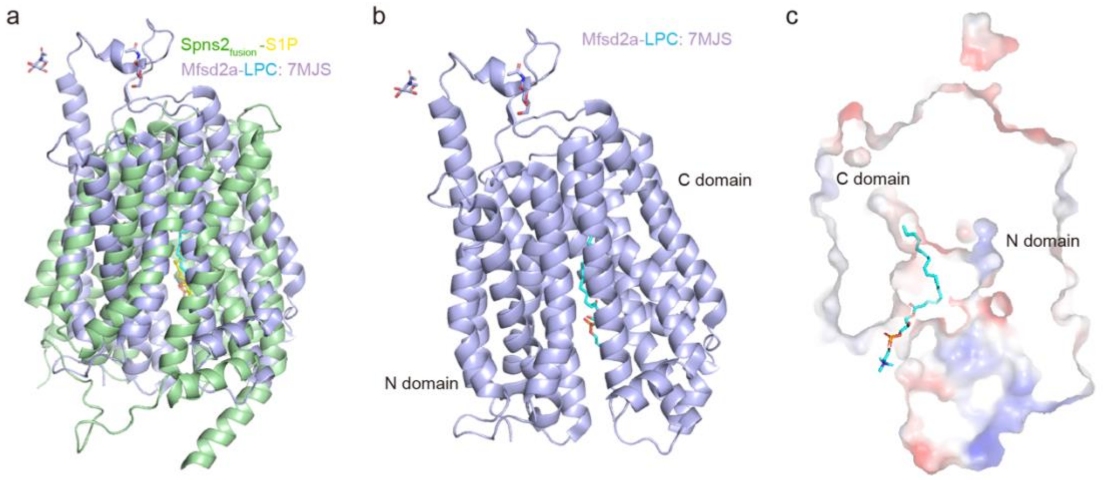

Fig. S8 Comparison between Spns2$_{fusion}$ and the LPC transporter MFSD2A

a. Superposition of Spns2$_{fusion}$ (pale green) with an inward-open structure of MFSD2A (PDB ID: 7MJS) (slate)**. b**. The inward-open structure of MFSD2A (PDB ID: 7MJS) (slate). An LPC molecule binds at the CTD side in the central cavity. LPC is shown as sticks and colored cyan. **c.** LPC in the substrate binding pocket of a cut view. The central cavity is shown with electrostatic potential.

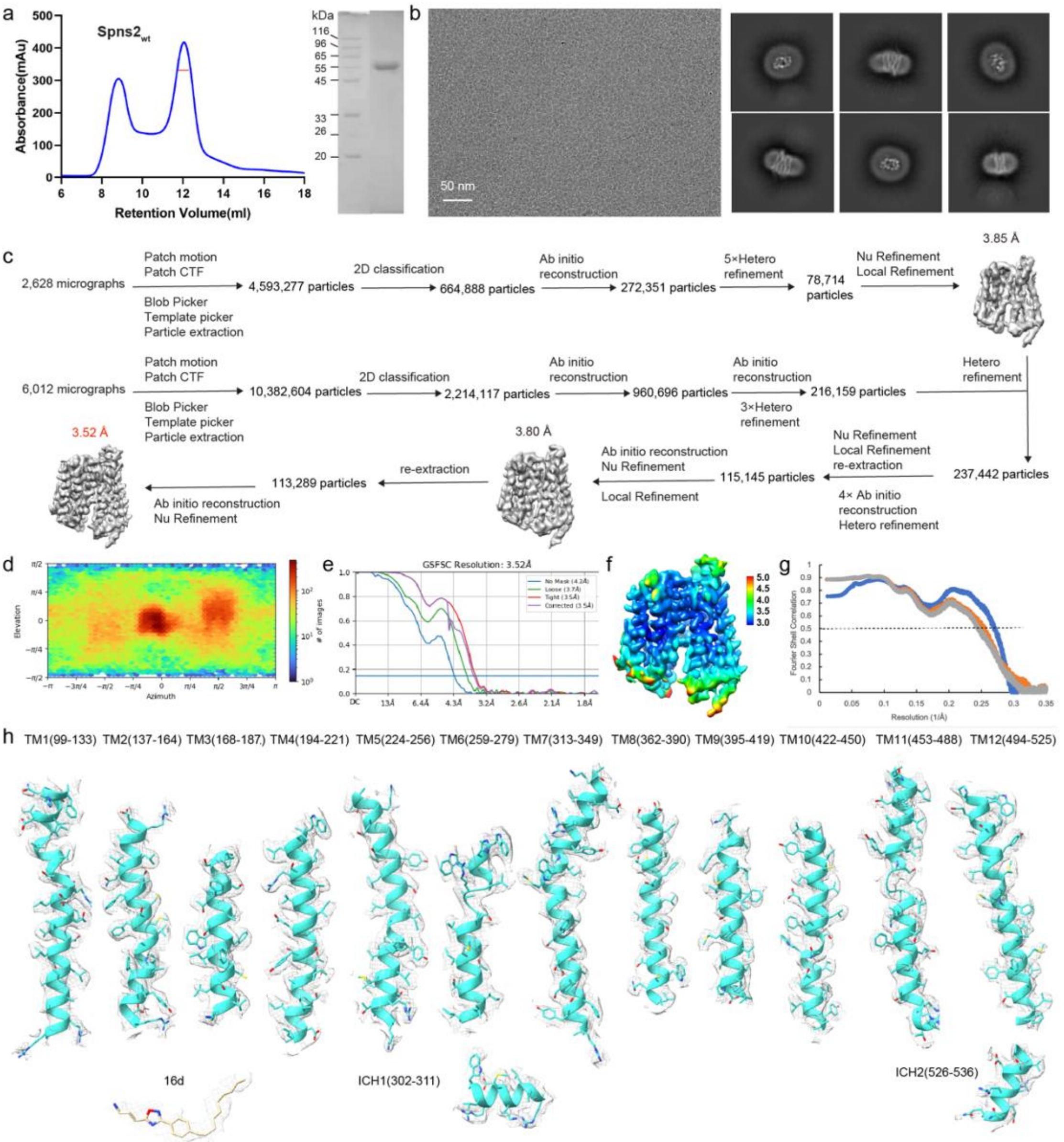

Fig. S9 Structural determination of Spns2wt

a. Spns2wt purification. Final size exclusion chromatography (Superdex 200 10/300 Increase) elution profile of Spns2wt. SDS-PAGE of one fraction at the elution peak is shown. **b**. Representative micrograph and 2D classes. **c.** Flowchart for EM data processing. Details can be found in the Methods. Representative 2D classes are displayed. **d**. Angular distribution of the particles used for the final reconstructions. **e.** Fourier shell correlation (FSC) curves of the final refined map. **f.** Local resolution of the final refined map of Spns2wt estimated by cryoSPARC. **g.** Fourier shell correlation (FSC) between map and model of Spns2wt. FSC curve of the final refined model against

the full map, colored in blue. FSC curve of the model refined against the first half map against the same map, colored in orange. FSC curve of the model refined against the first half map against the second half map, colored in gray. **h.** The density maps of the transmembrane and intracellular helices of $Spns2_{wt}$ are shown as mesh at a contour level of 0.2. The density maps of 16d are shown as mesh at a contour level of 0.1.

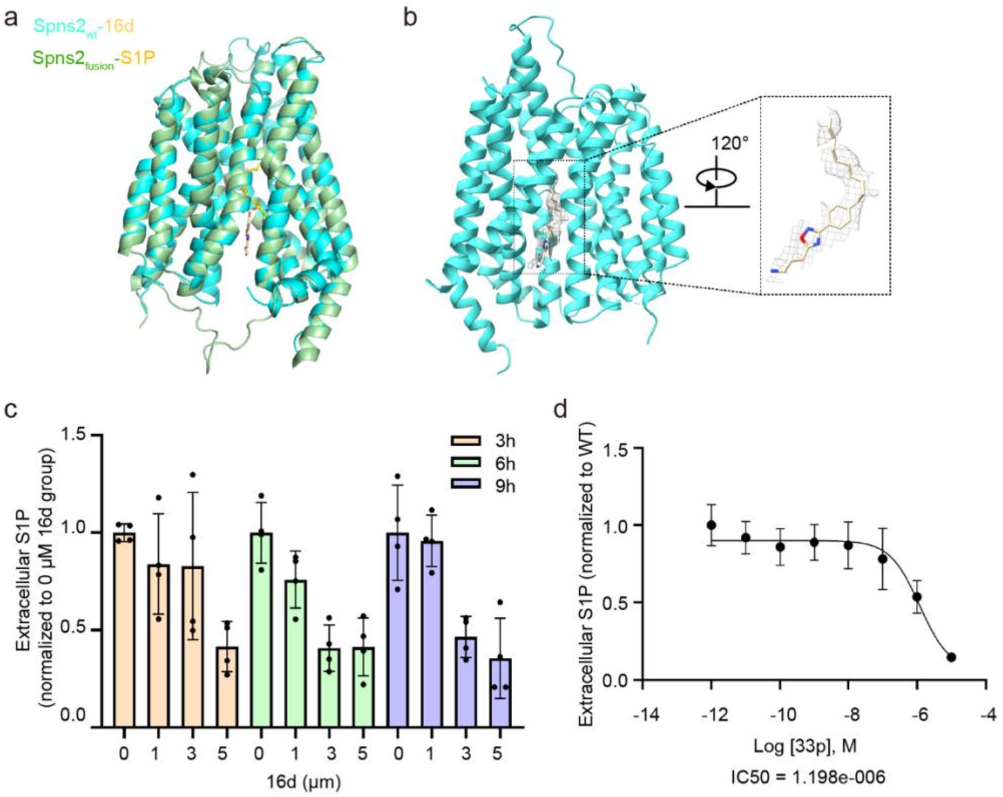

Fig. S10 Structure of Spns2$_{wt}$ binding with an inhibitor 16d

a. Superposition of Spns2$_{wt}$-16d (cyan) with Spns2$_{fusion}$-S1P (pale green)**.** The r.m.s.d. between the two structures is 1.458 Å over 421 Ca atoms. **b**. Inward-open structure of Spns2$_{wt}$ binding with an inhibitor 16d. Spns2$_{wt}$ is colored cyan. 16d density is shown in mesh at a contour level of 0.1. 16d is shown as sticks and colored wheat. A magnified view of 16d is displayed on the right. **c.** Inhibition of S1P release by 16d using HEK293T cells overexpressed Spns2. Data are presented as mean ± SD. n=2 biological replicates. **d**. The IC_{50} measurement of the new Spns2 inhibitor 33p. Data are represented as mean ± SD (n = 4 biological replicates).

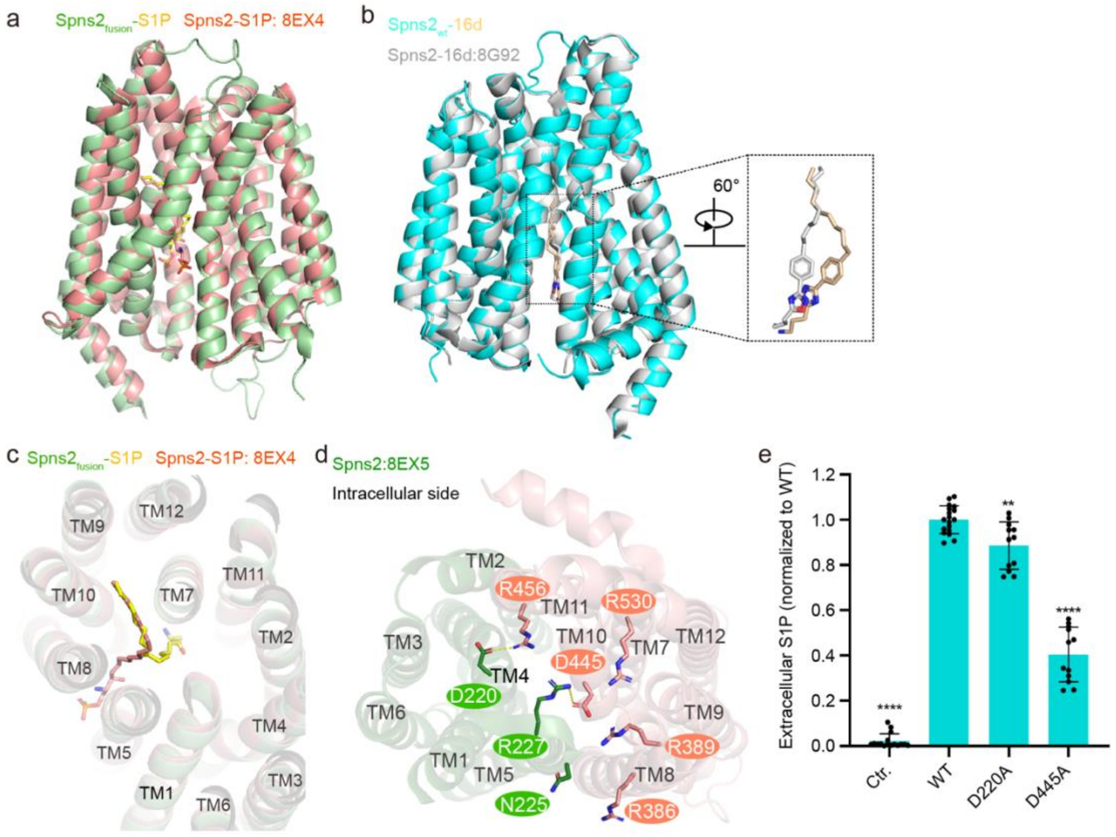

Fig. S11 Structure comparison with three reported structures

a. Superposition of Spns2fusion-S1P (pale green) with Spns2wt-S1P (PDB ID:8EX4) (salmon)**.** S1P is shown as sticks. **b**. Superposition of Spns2wt-16d (Spns2: cyan; 16d: wheat) with Spns2-16d (PDB ID:8G92) (gray)**.** 16d is shown as sticks. A magnified view of 16d is displayed on the right. **c**. Different binding site of the head part of S1P in Spns2fusion-S1P (pale green) and Spns2wt-S1P (PDB ID:8EX4) (salmon)**.** S1P is shown as sticks. **d.** Charge network at the intracellular side of the Spns2 outward-open structure (PDB ID:8EX5). Residues are shown as sticks. **e.** Transport assay of extracellular S1P level of overexpressing wild-type and mutations of residues (D220 and D445) in HEK293T cells. Data are presented as mean ± SD. n ⩾ 6 biological replicates; $p < 0.0001$(****).

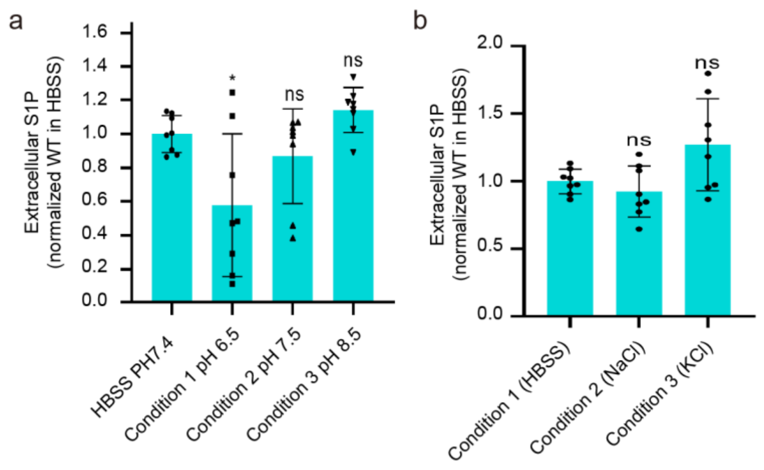

Fig. S12 Investigations of whether Spns2 is a cation or proton-coupled symporter or uniporter using cell-based transport assay

a. The transport activity of Spns2 under different pH conditions: Hank's balanced salt solution, HBSS (pH 7.4) and condition 1 (140 mM NaCl, 20 mM MES pH 6.5, 2 mM $CaCl_2$, 1 g/L D-glucose); Condition 2 (140 mM NaCl, 20 mM HEPES-NaOH pH 7.5, 2 mM $CaCl_2$, 1 g/L D-glucose); Condition 3 (140 mM NaCl, 20 mM Tris-HCl pH 8.5, 2 mM $CaCl_2$, 1 g/L D-glucose). Data are represented as mean ± SD (n = 4 biological replicates). **b.** The transport activity of Spns2 under different ionic conditions: condition 1 (Hank's balanced salt solution, HBSS); Condition 2 (140 mM NaCl, 20 mM Tris-HCl pH 7.5, 2 mM $CaCl_2$, 1 g/L D-glucose); Condition 3 (140 mM KCl, 20 mM Tris-HCl pH 7.5, 2 mM $CaCl_2$, 1 g/L D-glucose). Data are represented as mean ± SD (n = 4 biological replicates).

Table S1 Cryo-EM data collection, refinement, and validation statistics

	$Spns2_{fusion}$-S1P (EMDB-36284) (PDB 8JHQ)	$Spns2_{wt}$-16d (EMDB-36285) (PDB 8JHR)
**Data collection and processing**		
Magnification	105,000	105,000
Voltage (kV)	300	300
Electron exposure (e–/Å^2)	50.6-59.2	50.35; 49.88
Defocus range (μm)	-1.0-1.8	-1.5-2.0
Pixel size (Å)	0.85	0.425
Symmetry imposed	C1	C1
Initial particle images (no.)	7,397,481	14,975,881
Final particle images (no.)	390,253	113,289
Map resolution (Å)	3.6	3.52
FSC threshold	0.143	0.143
Map resolution range (Å)	3.0-5.0	3.0-5.0
**Refinement**		
Initial model used (PDB code)	N/A	N/A
Model resolution (Å)	3.5/3.8	3.4/3.7
FSC threshold	0.143/0.5	0.143/0.5
Model resolution range (Å)	N/A	N/A
Map sharpening *B* factor (Å^2)	N/A	N/A
Model composition		
Non-hydrogen atoms	3420	3302
Protein residues	446	430
Ligands	1	1
B factors (Å^2)		
Protein	90.54	86.30

Ligand	91.89	80.13
R.m.s. deviations		
Bond lengths (Å)	0.003	0.003
Bond angles (°)	0.594	0.697
Validation		
MolProbity score	1.71	1.67
Clashscore	7.93	10.96
Poor rotamers (%)	0	0.29
Ramachandran plot		
Favored (%)	95.93	97.42
Allowed (%)	4.07	2.58
Disallowed (%)	0	0

Table S2 Statistics of S1P transport assay and two-heart rescue assay in zebrafish

Samples	Transport activity (% wt)	Mean ±SD	Two-heart ratio (%wt)	N value
$Spns2_{wt}$[a]	100.00%	1 .000±0.012	4.05%	148
$Spns2_{wt}$[b]	100.00%	1 .000±0.006	4.28%	544
Ctr.[a]	0.56%	0.006±0.001	84.81%	237
Ctr.[b]	6.00%	0.060±0.016	81.65%	670
R200S	6.04%	0.060±0.008	86.96%	208
R227A-R456A	14.27%	0.143±0.016	72.35%	207
R342A	24.63%	0.246±0.026	22.00%	236
D137A	29.26%	0.293±0.013	70.07%	224
D128A	32.14%	0.321±0.020	63.33%	223
R119A	33.31%	0.333±0.024	38.62%	228
D137A-R342A	39.35%	0.394±0.020	51.86%	226
R227A	40.39%	0.404±0.053	63.59%	143
Y246A	42.71%	0.427±0.031	94.09%	203
S326A-Q463A-S464A	49.25%	0.493±0.026	56.27%	279
I429A	50.52%	0.505±0.017	66.77%	316
E207A	50.87%	0.509±0.035	25.61%	162
E207Q	52.72%	0.527±0.020	-	-
D118A	56.02%	0.560±0.044	50.27%	231
R456A	83.16%	0.832±0.051	38.84%	120

Cell based efflux assay data are presented as transport activity (% wt) and means ± SD. n≥4 biological replicates. Ctr. stands for the system control without Spns2 expression. WT and Ctr. for different graphs are listed separately. [a]Data for Fig. 1e-f. [b]Data for Fig. 1j-k. Two-heart rescue assay data in the zebrafish are presented as two heart ratios. N value is the replicate number of embryos.

Table S3 Statistics of S1P transport assay

Samples	Transport activity (% wt)	Mean ±SD
$Spns2_{wt}$[a]	100.00%	1 .000±0.039
$Spns2_{wt}$[b]	100.00%	1 .000±0.007
$Spns2_{wt}$[c]	100.00%	1 .000±0.010
$Spns2_{wt}$[d]	100.00%	1 .000±0.014
Ctr.[d]	2.15%	0.022 ±0.008
Ctr.[c]	2.30%	0.023 ±0.008
Ctr.[b]	5.10%	0.051 ±0.018
Ctr.[a]	6.15%	0.062 ±0.030
H468A	30.43%	0.304±0.044
D445A	40.32%	0.403±0.035
E433A	54.74%	0.547±0.026
D472A	55.06%	0.551±0.032
W440A	62.08%	0.621±0.032
N112A	67.07%	0.671±0.056
Q144A	68.45%	0.685±0.032
S326A-S464A	70.84%	0.708±0.031
L332A	73.48%	0.735±0.054
Y116A	75.37%	0.754±0.041
S326A-Q463A	75.79%	0.758±0.049
I336A	81.21%	0.812±0.054
F437A	87.42%	0.874±0.030
$Spns2_{fusion}$	87.54%	0.875±0.043
D220A	88.60%	0.886±0.030
Q463A-S464A	89.70%	0.897±0.022
Y235A	97.22%	0.972±0.032
Y120A	101.40%	1.014±0.030

Cell based efflux assay data are presented as transport activity (% wt) and means ± SD. n⩾4 biological

replicates. WT and Ctr. for different graphs are listed separately. [a]Data for fig. S3c. [b]Data for fig. S5h. [c]Data for fig. S5i. [d]Data for fig. S11e.

Table S4 Statistics of FTY720-P transport assay

Samples	Transport activity(% wt)	Mean ±SD
Spns2$_{wt}$	100.00%	1 .000±0.017
R119A	36.19%	0.362±0.018
D128A	36.52%	0.365±0.011
Y246A	38.26%	0.383±0.011
D137A-R342A	46.28%	0.463±0.011
R227A-R456A	50.05%	0.501±0.010
I429A	70.75%	0.708±0.010

Cell based efflux assay data are presented as transport activity (% wt) and means ± SD. n=4 biological replicates.
